# Effects of Absolute Humidity, Relative Humidity, Temperature, and Wind Speed on Influenza Activity in Toronto, Ontario, Canada

**DOI:** 10.1128/AEM.02426-18

**Published:** 2019-03-06

**Authors:** Adriana Peci, Anne-Luise Winter, Ye Li, Saravanamuttu Gnaneshan, Juan Liu, Samira Mubareka, Jonathan B. Gubbay

**Affiliations:** a Public Health Ontario, Toronto, Ontario, Canada; b Sunnybrook Research Institute, Toronto, Ontario, Canada; c University of Toronto, Toronto, Ontario, Canada; Centers for Disease Control and Prevention

**Keywords:** absolute humidity, relative humidity, temperature, wind speed, environmental factors, influenza

## Abstract

The occurrence of influenza in different climates has been shown to be associated with multiple meteorological factors. The incidence of influenza has been reported to increase during rainy seasons in tropical climates and during the dry, cold months of winter in temperate climates. This study was designed to explore the role of absolute humidity (AH), relative humidity (RH), temperature, and wind speed (WS) on influenza activity in the Toronto, ON, Canada, area. Environmental data obtained from four meteorological stations in the Toronto area over the period from 1 January 2010 to 31 December 2015 were linked to patient influenza data obtained for the same locality and period. Data were analyzed using correlation, negative binomial regressions with linear predictors, and splines to capture the nonlinear relationship between exposure and outcomes. Our study found a negative association of both AH and temperature with influenza A and B virus infections. The effect of RH on influenza A and B viruses was controversial. Temperature fluctuation was associated with increased numbers of influenza B virus infections. Influenza virus was less likely to be detected from community patients than from patients tested as part of an institutional outbreak investigation. This could be more indicative of nosocomial transmission rather than climactic factors. The nonlinear nature of the relationship of influenza A virus with temperature and of influenza B virus with AH, RH, and temperature could explain the complexity and variation between influenza A and B virus infections. Predicting influenza activity is important for the timing of implementation of disease prevention and control measures as well as for resource allocation.

**IMPORTANCE** This study examined the relationship between environmental factors and the occurrence of influenza in general. Since the seasonality of influenza A and B viruses is different in most temperate climates, we also examined each influenza virus separately. This study reports a negative association of both absolute humidity and temperature with influenza A and B viruses and tries to understand the controversial effect of RH on influenza A and B viruses. This study reports a nonlinear relation between influenza A and B viruses with temperature and influenza B virus with absolute and relative humidity. The nonlinear nature of these relations could explain the complexity and difference in seasonality between influenza A and B viruses, with the latter predominating later in the season. Separating community-based specimens from those obtained during outbreaks was also a novel approach in this research. These findings provide a further understanding of influenza virus transmission in temperate climates.

## Abstract (precis)

This study examined the relationship between environmental factors and the occurrence of influenza in general. Since the seasonality of influenza A and B viruses is different in most temperate climates, we also examined each influenza virus separately. This study reports a negative association of both absolute humidity and temperature with influenza A and B viruses and tries to understand the controversial effect of RH on influenza A and B viruses. This study reports a nonlinear relation between influenza A and B viruses with temperature and influenza B virus with absolute and relative humidity. The nonlinear nature of these relations could explain the complexity and difference in seasonality between influenza A and B viruses, with the latter predominating later in the season. Separating community-based specimens from those obtained during outbreaks was also a novel approach in this research. These findings provide a further understanding of influenza virus transmission in temperate climates.

## INTRODUCTION

The worldwide occurrence and detection of influenza and other respiratory viruses in different climates have been shown to be associated with multiple environmental factors. The incidence of influenza has been reported to increase during rainy seasons in tropical climates and during the dry, cold months of winter in temperate climates (1, 2). The root causes of these phenomena are not well understood. In temperate climates, various studies have suggested that low indoor and outdoor humidity, typically found in winter, contributes to improving the survival of viruses in droplets (3, 4), thus facilitating the transmission of influenza virus. Humidity is also believed to affect the salt content of droplets; at high relative humidity (RH) (i.e., 99% to 100%, or physiological conditions), viruses tend to stay stable as physiological salt concentrations are maintained (5), while at lower RH (∼50% to 99%), the increase in the saline concentration due to evaporation may lead to damage to the influenza virus. At the lowest RH (<50%), salts get crystallized and the stability of the viruses is maintained. Previous studies have explored the role of both RH and absolute humidity (AH) on influenza virus survival and transmission, concluding that AH had better control over influenza virus survival and transmission than RH (3, 6–8). Another environmental factor postulated to affect influenza virus transmission is wind speed (WS). A study examining the transmission of influenza virus under field conditions reported that a wind speed of >30 km/h from the direction of nearby infected premises was associated with an increased hazard ratio of influenza virus infection in horses (9). The authors speculated that sufficient aerosols coughed by an infected individual could travel longer if they were assisted by the wind, transmitting the infection to another individual. Wind also lowers the outdoor temperature and further dries the air, both of which are reported to facilitate influenza virus transmission (10). In addition, a variety of host factors, such as inhibition of the immune system, potentially through the decreased levels of vitamin D production in winter (2) and the reduction in the clearance ability of respiratory cilia with the inhalation of cold air (11, 12), have also been reported to enhance the transmission of influenza virus during winter seasons.

Further, it has also been reported from animal (3) and human (13) studies that in temperate climates influenza virus transmission is more likely to occur at temperatures of 5°C and below. Other studies have noted that the incidence of acute viral respiratory tract infections may be related to fluctuations in temperature rather than colder temperatures on their own (1, 13). Relatively warm temperatures along with higher humidity followed by a decline in both factors have also been reported to increase the risk of influenza virus infection (13).

In addition to the virus being more stable at low temperatures and low humidity, cold weather leads to indoor crowding, which facilitates virus transmission through particle droplets generated by coughing and sneezing among those in close contact (4, 11, 14, 15). Indoor crowding may explain some of the higher attack rates seen in institutional outbreaks. Other studies have reported that the impact of any individual environmental factor may be low in the calculation of the total factors contributing to the incidence of influenza virus infection. In one study, the impact of absolute humidity on influenza virus was reported to be about 3%, and any effects of humidity were postulated to be synergistic with other environmental and host factors (16).

While influenza activity in temperate climates primarily occurs between late fall and early spring, there is often intra- and interseasonal variability in its timing, the causative virus type, and seasonal severity. For example, in Ontario, Canada, influenza A (H3N2) virus-dominant seasons often commence in November, while influenza B virus-dominant seasons start later, usually in February (17).

Discerning the specific factors that facilitate influenza virus transmission is fraught with difficulties due to the presence of multiple potential confounders. As pointed out by Roussel et al., exploring the interactions between various climactic conditions and influenza incidence would be more realistic in nature than exploring overly simplistic relationship between disease incidence and individual conditions (2).

This study was designed to explore the role of environmental factors, including AH, RH, WS, temperature, and temperature fluctuation, on influenza activity in Toronto, ON, Canada, from 1 January 2010 to 31 December 2015. Specifically, this study was aimed at exploring and comparing the strength of the association between AH and RH with influenza occurrence. Data from sporadically occurring as well as outbreak-associated laboratory-confirmed cases were included to explore the potential effects of nosocomial transmission in an institution.

## RESULTS

### Seasonality of influenza.

Influenza A virus peaked during the winter months in Toronto, whereas influenza B virus peaked later, in early spring.

**FIG 1:**
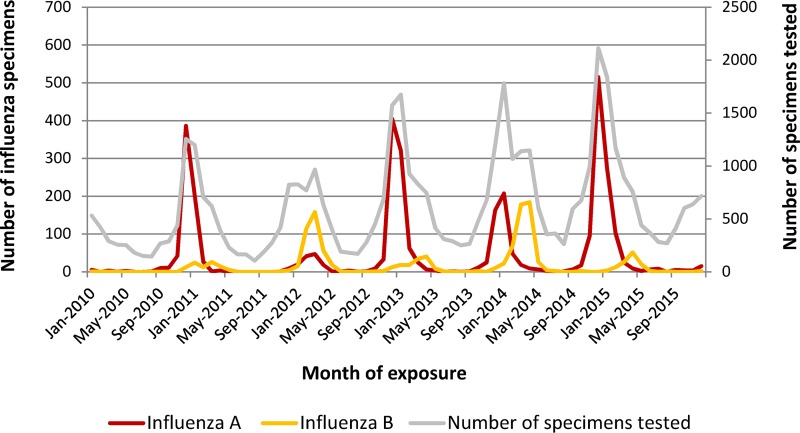
Epidemic curve of influenza A and B virus activity by month of exposure, Toronto area, January 2010 to December 2015. Influenza A virus activity peaked during the December to January period, while influenza B virus activity peaked later in the season (March to April).

A total of 44,362 patient specimens were tested for influenza virus and included in this study. All patients were from the Toronto area. The median age of the patients was 63 years, with the age range being from 1 month to 108 years. Those who tested positive for influenza virus were slightly older; the median age was 67 years, and the age range was 2 months to 107 years (*P* < 0.001). Of all specimens included in this study, a total of 4,485 (10%) specimens were positive for influenza virus. Specifically, 3,275 and 1,210 specimens were positive for influenza A virus and influenza B virus, respectively. December to January was the period when most influenza A virus-positive specimens were detected (2,521 [76.9%] of all influenza A virus-positive specimens), whereas March to April was the period with the highest number (767, 63.6%) of influenza B virus detections (Fig. 1). Of all specimens tested, 40,311 were submitted from physicians’ offices or hospitals (inpatient wards, intensive care units, or emergency departments) and 4,051 (9.1%) specimens were submitted from institutional respiratory outbreaks. Of all outbreak-related specimens, 1,140 (28.1%) were positive for influenza virus.

### Climate in Toronto.

The median and range for each environmental factor for daily, weekly, and monthly durations are presented in Table 1.

**TABLE 1:** Daily, weekly, and monthly values for environmental factors in Toronto area, January 2010 to December 2015^*a*^

Time interval	AH (g/m^3^)	RH (%)	Temp (°C)	WS (km/h)
Daily	6.2 (0.5 to 20.7)	71.1 (31.5 to 99.5)	9.4 (−20.7 to 31.1)	13 (4.01 to 42.1)
Weekly	6.0 (1.0 to 17.8)	70.2 (40.0 to 90.0)	9.3 (−16.9 to 27.3)	13 (6.0 to 28.0)
Monthly	6.2 (1.4 to 16.4)	70.8 (51.5 to 82.1)	9.9 (−10.4 to 23.8)	13 (9.0 to 19.0)

a The values represent the median (range). AH, absolute humidity; RH, relative humidity; WS, wind speed.

**FIG 2:**
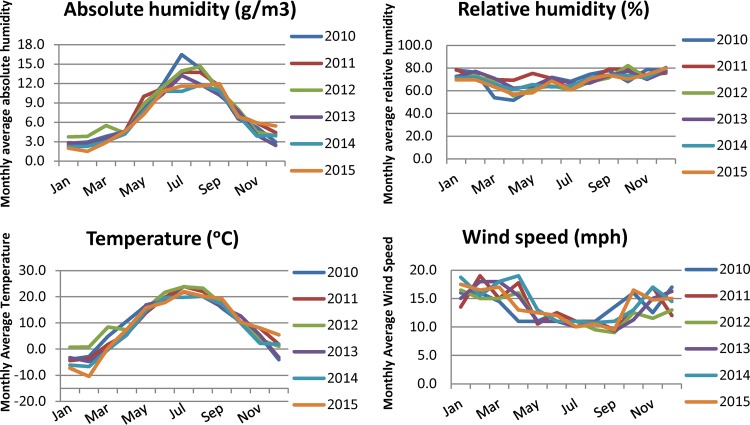
Absolute humidity, relative humidity, temperature, and wind speed in the Toronto area, January 2010 to December 2015. AH and temperature were the highest in the summer and the lowest in the winter, whereas RH and WS did not vary considerably between months.

AH and temperature were the highest in summer (July) and the lowest in winter (January and February, respectively), whereas RH and WS did not vary considerably between months (Fig. 2).

### Correlation analyses: only AH was highly correlated with temperature.

Correlation analyses between environmental factors showed a strong positive linear association between AH and temperature, with a Pearson correlation coefficient of 0.91 (Table 2); none of the other correlations was statistically significant. Correlation analyses between each environmental factor and overall influenza virus, influenza A virus, influenza A/H3N2 virus subtype, and influenza B virus activity demonstrated weak associations.

**TABLE 2:** Correlation analyses exploring the association within and between environmental factors and detection of influenza types and subtypes^*a*^

Variable	Pearson’s correlation coefficient				
	AH	RH	Temp	WS	Temp fluctuation
RH	0.08				
Temp	0.91	−0.03			
WS	−0.38	−0.10	−0.38		
Temp fluctuation	0.15	0.01	0.19	−0.19	
Influenza A virus	−0.33	0.24	−0.28	0.26	−0.06
Influenza A/H3N2 virus	−0.35	0.26	−0.4	0.19	−0.08
Influenza B virus	−0.26	−0.20	−0.45	0.26	0.11
All influenza viruses	−0.27	0.07	−0.31	0.19	−0.01

a AH, absolute humidity; RH, relative humidity; WS, wind speed. Weekly medians were used to measure AH, RH, temperature, WS, and temperature fluctuation. Pearson’s correlation coefficients range from −1 to 1, with negative values indicating a negative association and positive values indicating a positive association. The strength of the association is interpreted as weak when coefficients are in the range of 0.1 to 0.3, medium when the coefficients are in the range of 0.4 to 0.7, and strong when the coefficients are in the range of 0.7 to 1.0. Temperature fluctuation was calculated as the median daily temperature of the assumed exposure date minus the median temperature on the previous day. The weekly median of temperature fluctuation was used for this analysis. Influenza A and B viruses represent the weekly total counts of influenza A and B virus-positive specimens. Influenza A virus represents the sum of the weekly counts of both influenza A and B viruses. Analyses for influenza A/H3N2 virus were restricted to influenza A virus-positive specimens that had subtyping performed.

### Linear negative binomial regression models.

There was a negative association between AH and temperature with influenza activity. A contrasting effect of RH with the influenza virus type was observed.

The results of AH and RH linear negative binomial models adjusted for other environmental factors and the interaction effect between temperature and temperature fluctuation, patient age, and outbreak status are presented in Tables 3 and 4. An increase in AH or temperature was associated with a decrease in overall influenza virus and influenza A and B virus detections (Table 3). An increase in RH was associated with an increase in influenza A virus detection and a decrease in influenza B virus detection (Table 4). No association was found between WS and any of the influenza virus detection outcomes. An increase in temperature fluctuation was associated with an increase in overall influenza virus detection and, specifically, influenza B virus detection. Both influenza A and influenza B viruses were significantly less likely to be detected from community-based specimens than from outbreak-associated specimens. The interaction effect was at borderline significance for overall influenza virus and influenza B virus detections and was not significant for influenza A virus detection. Comparing the effect of AH and RH with all influenza virus detections, a 1-unit increase in the weekly median AH or RH was associated with a 20% or 1% decrease, respectively, in all influenza virus counts, with the value for RH being at borderline significance (Tables 3 and 4).

**TABLE 3:** Adjusted AH linear negative binomial regression models exploring the relationship of environmental factors with the number of specimens positive for influenza viruses overall and influenza A and B viruses^*a*^

Demographic or climatic factor	Adjusted IRR (95% CI) for linear AH model		
	All influenza viruses	Influenza A virus	Influenza B virus
Age group (yr)			
65+	1.00	1.00	1.00
<1	1.34 (1.01–1.78)*	1.79 (1.23–2.60)*	0.71 (0.41–1.22)
1–4	2.88 (2.19–3.79)*	3.39 (2.35–4.89)*	1.80 (1.08–3.00)*
5–19	6.76 (4.88–9.36)*	7.19 (4.62–11.1)*	4.32 (2.37–7.88)*
20–64	1.66 (1.38–2.01)*	1.92 (1.48–2.48)*	1.26 (0.89–1.78)
Outbreak status			
Yes	1.00	1.00	1.00
No	0.11 (0.09–0.15)*	0.07 (0.05–0.11)*	0.27 (0.16–0.46)*
AH	0.80 (0.78–0.83)*	0.79 (0.76–0.82)*	0.80 (0.76–0.84)*
WS	1.00 (0.98–1.02)	0.97 (0.95–1.00)	1.03 (0.99–1.07)
Temp fluctuation	1.03 (1.01–1.04)*	0.99 (0.97–1.02)	1.07 (1.04–1.10)*
Temp · temp fluctuation	1.00 (1.00–1.01)*	1.00 (0.99–1.00)	1.00 (1.00–1.00)*

a AH, absolute humidity; RH, relative humidity; WS, wind speed; IRR, incidence relative risk, CI, confidence interval. The weekly median was used to measure AH, RH, WS, and temperature. The estimate is considered significant (*) when the 95% confidence interval does not cross 1. The adjusted incidence relative risk of 1.00 indicates the category used as a reference/comparison. The total weekly numbers of positive influenza A and B virus counts were used as dependent variables. The left column indicates all independent/predictable variables for which this model was adjusted. Temperature fluctuation represents the difference in the median temperatures between the exposure day and the previous day. In this model, the weekly median of this measurement was included. Temperature · temperature fluctuation represents the interaction term for which the model was adjusted. These models include measurements of climatic factors and detection of influenza A and B viruses and all influenza virus-positive specimens for 293 unique weeks.

**TABLE 4:** Adjusted RH linear negative binomial regression models exploring the relationship of environmental factors with detection of influenza A and B viruses and all influenza viruses^*a*^

Demographic or climatic factor	Adjusted IRR (95% CI) for the linear RH model		
	All influenza viruses	Influenza A virus	Influenza B virus
Age group (yr)			
65+	1.00	1.00	1.00
<1	1.29 (0.96–1.73)	1.36 (0.94–1.97)*	1.01 (0.59-1.72)
1–4	2.8 (2.11–3.71)*	2.55 (1.78–3.65)*	2.76 (1.66-4.58)*
5–19	6.49 (4.63–9.10)*	4.80 (3.11–7.42)*	6.98 (3.83-12.7)*
20–64	1.65 (1.36–1.99)	1.69 (1.32–2.17)*	1.49 (1.06-2.10)*
Outbreak status			
Yes	1.00	1.00	1.00
No	0.12 (0.09–0.16)*	0.11 (0.07–0.15)*	0.19 (0.11-0.32)*
RH	0.99 (0.98–1.00)	1.03 (1.02–1.04)*	0.94 (0.93–0.95)*
Wind speed	1.01 (0.99–1.04)	1.00 (0.97–1.03)	1.03 (1.00–1.07)
Temp	0.93 (0.92–0.94)*	0.91 (0.90–0.93)*	0.94 (0.93–0.96)*
Temp fluctuation	1.04 (1.02–1.05)*	0.99 (0.97–1.02)	1.09 (1.05–1.12)*
Temp · temp fluctuation	1.00 (1.00–1.00)*	1.00 (0.99–1.00)	1.00 (0.99–1.00)

a AH, absolute humidity; RH, relative humidity; WS, wind speed; IRR, incidence relative risk, CI, confidence interval. The weekly median was used to measure AH, RH, WS, and temperature. The estimate is considered significant (*) when the 95% confidence interval does not cross 1. An incidence relative risk of 1.00 indicates that the category was used as a reference/comparison. The total weekly numbers of positive influenza A and B counts were used as dependent variables. The left column indicates all independent/predictable variables for which this model was adjusted. Temperature fluctuation represented the difference in median temperatures between the exposure day and the previous day. In this model, the weekly median of this measurement was included. Temperature · temperature fluctuation represents the interaction term for which the model was adjusted. These models include measurement of climatic factors and detection of influenza A and B viruses and all influenza virus-positive specimens for 293 unique weeks.

Negative binomial regression analyses found no association between detection of the influenza A/H3N2 virus subtype and environmental factors. However, these results should be interpreted with caution, as these analyses were restricted only to influenza A virus-infected specimens with complete subtype testing.

### Negative binomial regression models with splines.

A nonlinear relationship of temperature with both influenza A and B virus detection was confirmed. A nonlinear relationship of AH and RH with only influenza B virus detection was also confirmed.

The results of nonlinear AH and RH negative binomial regression models adjusted for other environmental factors, patient age, and outbreak status are presented in Tables 5 and 6.

**TABLE 5:** Adjusted AH nonlinear negative binomial regression models exploring the relationship of environmental factors with influenza activity and the nonlinearity of AH and temperature with influenza A and B viruses^*a*^

Demographic or climatic factor	All influenza viruses			Influenza A virus			Influenza B virus		
	IRR (95% CI)	*P* value	Nonlinearity *P* value	IRR (95% CI)	Association *P* value	Nonlinearity *P* value	IRR (95% CI)	*P* value	Nonlinearity *P* value
Age group (yr)									
65+	1.00	NA	NA	1.00	NA	NA	1.00	NA	NA
<1	0.69 (0.55–0.87)	<0.0001*	NA	0.70 (0.52–0.95)	0.0016*	NA	0.70 (0.47–1.04)	<0.0001	NA
1–4	1.35 (1.10–1.67)	<0.0001*	NA	1.21 (0.92–1.61)	<0.0001	NA	1.67 (1.18–2.37)	<0.0001*	NA
5–19	2.43 (1.97–3.01)	<0.0001*	NA	1.78 (1.32–2.39)	0.0007*	NA	4.07 (2.89–5.72)	0.004*	NA
20–64	1.19 (1.00–1.41)	0.002	NA	1.20 (0.96–1.50)	0.0243	NA	1.26 (0.95–1.69)	0.084	NA
Outbreak status									
Yes	1.00	NA	NA	1.00	NA	NA	1.00	NA	NA
No	0.26 (0.23–0.31)	<0.0001*	NA	0.24 (0.19–0.30)	<0.0001*	NA	0.31 (0.24–0.41)	<0.0001*	NA
AH^*b*^	NA	<0.0001*	0.0202*	NA	<0.0001*	0.2348	NA	<0.0001*	<0.0001
WS	1.00 (0.98–1.02)	0.82	NA	0.97 (0.94–1.00)	0.0824	NA	1.04 (1.00–1.08)	0.0152	NA
Temp fluctuation	1.03 (1.01–1.05)	<0.0001*	NA	1.00 (0.98–1.02)	0.4897	NA	1.07 (1.04–1.10)	<0.0001*	NA

a AH, absolute humidity; WS, wind speed; IRR, incidence relative risk, CI, confidence interval; NA, the measurement is not applicable for that variable. The AH nonlinear regression model explored the association of environmental factors with influenza activity as well as the nonlinearity of the association of AH with all influenza viruses and influenza A and B viruses. The left column lists independent/predictable variables for which this model was adjusted. The total weekly numbers of positive influenza A and B virus counts were used as dependent variables. A significant result (*) for association is considered when the 95% confidence interval does not cross 1 and the *P* value is <0.05. A significant result for nonlinearity is considered when the *P* value is <0.05. An incidence relative risk of 1.00 indicates the category used for reference/comparison. AH, temperature, and WS were measured by the use of weekly median measurements. Influenza A and influenza B viruses represent the total weekly numbers of positive specimens. All influenza viruses represent the sum of influenza A and B virus-positive specimens.

b AH was also examined for a nonlinear association with influenza A and B viruses.

**TABLE 6:** Adjusted RH nonlinear negative binomial regression models exploring the relationship of environmental factors and nonlinearity of RH with influenza A and B viruses^*a*^

Demographic or climatic factor	All influenza viruses			Influenza A virus			Influenza B virus		
	IRR (95% CI)	*P* value	Nonlinearity *P* value	IRR (95% CI)	Association *P* value	Nonlinearity *P* value	IRR (95% CI)	*P* value	Nonlinearity *P* value
Age group (yr)									
65+	1.00	NA	NA	1.00	NA	NA	1.00	NA	NA
<1	0.67 (0.53-0.85)	<0.0001*	NA	0.69 (0.50-0.95)	0.0024*	NA	0.73 (0.49-1.10)	<0.0001	NA
1–4	1.32 (1.07–1.63)	<0.0001*	NA	1.18 (0.88–1.59)	<0.0001	NA	1.89 (1.33–2.67)	<0.0001*	NA
5–19	2.42 (1.95–3.01)	<0.0001*	NA	1.82 (1.33–2.48)	0.005*	NA	4.32 (3.07–6.09)	0.0037*	NA
20–64	1.19 (1.01–1.42)	0.0012*	NA	1.22 (0.96–1.54)	0.0247	NA	1.33 (0.99–1.78)	0.1316	NA
Outbreak status									
Yes	1.00	NA	NA	1.00	NA	NA	1.00	NA	NA
No	0.27 (0.23–0.32)	<0.0001*	NA	0.23 (0.19–0.29)	<0.0001*	NA	0.30 (0.22–0.39)	<0.0001*	NA
RH^*b*^	NA	0.0013*	0.0056*	NA	<0.0001*	0.4923	NA	<0.0001*	<0.0001*
Temp	NA	<0.0001*	<0.0001*	NA	<0.0001*	<0.0001*	NA	<0.0001*	<0.0001*
WS	1.00 (0.98–1.02)	0.8200	NA	1.00 (0.97–1.03)	0.8879	NA	0.99 (0.95–1.02)	0.6061	NA
Temp fluctuation	1.03 (1.01–1.05)	<0.0001*	<0.0001	0.99 (0.97–1.01)	<0.0001	NA	1.09 (1.06–1.11)	<0.0001*	NA

a RH, relative humidity; WS, wind speed; IRR, incidence relative risk, CI, confidence interval; NA, the measurement is not applicable for that variable. The RH nonlinear regression model explored the association of environmental factors with influenza activity as well as the nonlinearity of the association for RH and temperature with influenza A and B viruses and all influenza viruses. The left column lists independent/predictable variables for which this model was adjusted. The total weekly numbers of positive influenza A and B virus counts were used as dependent variables. A significant result (*) for association is considered when the 95% confidence interval does not cross 1 and the *P* value is <0.05. A significant result for nonlinearity is considered when the *P* value is <0.05. The incidence relative risk of 1.00 indicates the category used for reference/comparison. AH, temperature, and WS were measured by the use of weekly median measurements. Influenza A virus and influenza B virus represent the total weekly numbers of positive specimens. All influenza viruses represent the sum of influenza A and B virus-positive specimens.

b RH was also examined for a nonlinear association with influenza A and B viruses.

In the nonlinear model, influenza A and B viruses were more likely to be detected among those younger than 65 years of age but older than 1 year of age than among those older than 65 years of age.

**FIG 3:**
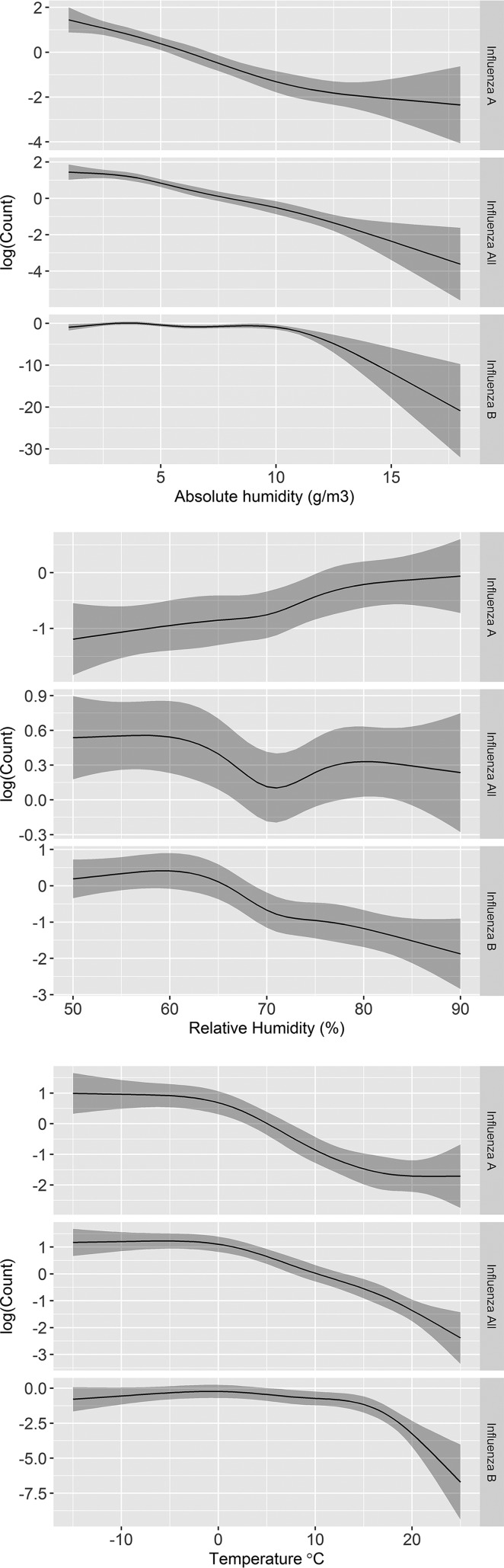
Adjusted nonlinear negative regression model examining the relationship of AH, RH, and temperature with overall influenza virus and influenza A and B virus detection. The dark gray shading represents the 95% confidence interval band. Nonlinearity was significant only for absolute and relative humidity with influenza B virus and for temperature with both influenza A and B viruses.

Nonlinearity was significant only for AH with influenza B virus (*P* < 0.001) (Table 5). An increase in AH was associated with a decrease in the detection of influenza viruses overall and, specifically, with a more consistent decrease in the detection of influenza A virus compared to influenza B virus (Fig. 3). Influenza B virus detection was constant until AH went above the 10.5-g/m^3^ threshold, which was associated with a dramatic decrease in the detection of influenza B virus.

In the nonlinear RH model, nonlinearity was significant only for RH with influenza B virus detection, and the association of RH with the detection of both influenza A and B viruses was significant (Table 6). An increase in RH was associated with an increase in influenza A virus detection and a decrease of influenza B virus detection only after RH went above 60% (Fig. 3); before that, influenza B virus detection was constant. The association of temperature with influenza virus detection was nonlinear for both influenza A or B viruses (Table 6). A negative association between temperature and the detection of both influenza A and B viruses was found. Specifically, an increase in temperature resulted in a rapid decrease in influenza A virus detection, particularly when the temperature was above 0°C, and in influenza B virus detection when the temperature went above 15°C (Fig. 3). The strength of the association for AH and RH with all influenza virus counts was significant for both nonlinear relations (*P* < 0.0001 and *P* < 0.0013, respectively) (Tables 5 and 6).

The nonlinear results for other independent variables were very similar to those reported from the linear models.

## DISCUSSION

In this study, we examined the relationships between environmental factors, such as AH, RH, temperature, temperature fluctuation, and WS, and influenza activity. Laboratory-confirmed detections of influenza virus in respiratory specimens were used as the measure of influenza activity. Environmental factors were measured from four meteorological stations in the Toronto area. Our study showed that influenza viruses were detected at a higher rate in individuals younger than 65 years but older than 1 year than in elderly individuals (age, 65+ years). This could be because younger people spend more time outdoors and elderly individuals and infants have limited access to the outdoors during low temperature and rainy weather (18). Younger patients also shed higher levels of virus when they are infected and also get tested earlier, which facilitates influenza virus detection (19).

A linear correlation between environmental factors and influenza A or B virus detection demonstrated weak associations, due to the fact that correlation is a limited application to measure the strength of the association between variables that have nonlinear complex relationships.

Nonlinear negative binomial regression demonstrated that as AH increased, influenza A virus detection steadily decreased, while for influenza B virus, the decrease occurred only when AH went above the 10.5-g/m^3^ threshold. Similarly, Shoji et al. reported that the start of influenza season in a temperate climate is when AH decreases to less than 5 g/m^3^ (8). Barreca and Shimshack reported a nonlinear relation between influenza and AH and a higher rate of mortality from influenza when AH levels are below 6 g/m^3^; however, they did not explore the association of AH by influenza virus type (20). A full understanding of the mechanism by which AH affects virus survival is still lacking. Yang et al. have suggested that higher levels of AH lead to surface inactivation of lipid-containing viruses due to the denaturing of virus lipoproteins (5). The mechanism for this is proposed to be related to phase changes of the phospholipid bilayer membrane leading to cross-linking of peptides at the air-water interface (21).

We found a positive relation between RH and influenza A virus detection, while influenza B virus detection decreased only after RH went above the 60% threshold. Similar to our study, Noti at al. reported that influenza virus remains five times more infectious at an RH interval of 7 to 23% than at an RH of 43% and above (22). It has been postulated that a low RH leads to evaporation of virus particles, thus allowing them to remain airborne for a longer period of time and increasing the opportunity to infect new hosts (10). We postulate that the contrasting effect of RH with influenza A and B virus detection might be due to the difference between outdoor and indoor RHs during winter. Studies have shown that while the outdoor RH peaks in winter, the indoor RH is the lowest due to the drying effect of heating systems, which may lead to the increased transmission of influenza A virus indoors (23). This difference between indoor and outdoor RHs may not apply to influenza B virus, which predominates later in the season, when the temperature gets warmer. AH might be a better predictor of influenza, as AH does not vary between the indoor and outdoor settings (24).

Similar to the effect of AH, we found a nonlinear effect of temperature on both influenza A and B virus detection; an increase in temperature was associated with a decrease in influenza virus detection only when the temperature went above 0°C for influenza A virus and above a 15°C threshold for influenza B virus (Fig. 3). This finding may explain the difference in seasonality between influenza A virus and influenza B virus, particularly because influenza B virus seems to predominate later in the season. Previous research reported that AH and temperature are driving factors for influenza activity (25). In addition, a decrease in both temperature and AH was reported to be associated with an increase in influenza virus detection (13).

Various hypotheses exist to explain associations between low temperatures and influenza virus detection, implicating virus survival, human immunity, and virus transmission. First, increased virion stability during cold temperatures is hypothesized to be related to the decreased activities of proteases (26). Second, lower temperatures are associated with a reduction of mucus and ciliary movement, leading to inhibition of mechanical defense and immunity toward infection (2). In addition, Horst et al. reported that the levels of several monocyte-derived cytokines peak in summer and are lower in winter (26). This may explain an important pathophysiological mechanism in the human immune response that affects host susceptibility and increases influenza virus transmission in winter (26). Lastly, low outdoor temperatures may result in indoor crowding, which facilitates virus transmission (12, 15). However, none of these theories are scientifically established. Further research is needed to understand the variability of these relations between influenza A and B virus activity.

We found that temperature fluctuation was associated with an increase in influenza B virus detection but not influenza A virus detection. The interpretation of this finding is complicated, as the association of temperature itself with influenza B virus detection was nonlinear. Influenza B virus infection cases dropped after the temperature went above 15°C; however, this drop was slower when it was associated with higher temperature fluctuation.

Previous studies reported that the incidence of influenza is related to fluctuations in temperature rather than low temperature *per se* (1). Other studies (10, 13, 22) reported that a decrease in temperature and humidity preceded the onset of influenza activity. The reason why we did not find the same effect might be due to the method used to calculate temperature fluctuation. We used the difference between the average temperature on the exposure day and that on the previous day and averaged it weekly, which might have smoothed the true effect. Makinen et al used the difference in the maximum and minimum temperatures over the 3 days before the exposure day (24), which could be more drastic and facilitate the detection of an effect but may not represent temperature fluctuations in reality.

Significantly less influenza A and B virus was detected from specimens tested from patients in community settings than from specimens tested as part of outbreak investigations. This might be due to testing bias, as outbreaks are reportable to public health authorities; thus, testing is mandated more often for outbreaks than for community patients, leading to lower rates of detection of influenza virus in the latter. However, if this effect is true, it is challenging to distinguish the contribution of external (i.e., environmental) factors from factors associated with indoor crowding and other human behaviors that may enhance virus transmission or host susceptibility in an outbreak setting. In addition, indoor heating during the wintertime makes the air drier, favoring the spread of influenza viruses by reducing the size of the aerosol particles through evaporation (27). Maintaining a higher level of humidity indoors in an outbreak setting might help in reducing transmission.

Some controversial evidence reported that low WS helps influenza (H1N1) virus transmission (28). We found no effect of WS on influenza activity; this may be due to the use of weekly averages, which may not be ideal.

Our study has a number of limitations. We were unable to examine the entire province of Ontario, as the meteorological stations were primarily located in the Toronto area. The exposure date may not necessarily represent the true date of disease exposure; however, we based the period of communicability and the incubation period on the scientific literature, and this time frame was applied for all samples. The specimens tested represented a small proportion of individuals ill with influenza, as many individuals do not seek medical attention, and thus, samples from these individuals are not eligible for laboratory testing.

Due to testing limitations, we were unable to fully explore the association of any of the influenza virus subtypes or strain types with environmental factors.

Further, we could not rule out the possible influence of several confounding factors, such as indoor crowding and heating, precipitation, and air pollution, that may have affected the influenza virus counts and further biased our results. The results of this study may be generalizable to other countries in the Northern Hemisphere with a temperate climate or even some tropical countries in the Southern Hemisphere that have distinct seasons like temperate climates. However, the results may not be generalizable to other countries with tropical or subtropical climates in which the influenza season is highly heterogeneous, lasts longer, and does not manifest a distinct seasonal pattern (29). Finally, we did not consider host factors, such as immunity or frailty, which play a role in disease transmission and also influence who is likely to be tested for influenza, nor did we consider seasonal influenza vaccine effectiveness.

Despite these limitations, our study has several strengths. First, we used laboratory-confirmed outcomes for influenza A or B virus detection. Previous studies examined the association of environmental factors with less specific diagnoses, such as influenza-like illness (ILI) or severe acute respiratory infection (SARI) (30). Similarly, they reported a negative association between temperature or AH and ILI and between RH and SARI but a positive relation between a temperature increase and SARI (30). The authors speculate that the latter could have been due to higher air pollution levels in warm weather or increased rainfall due to oscillation between warm and cold weather. This could also be due to the circulation of non-influenza virus respiratory viruses, such as enterovirus/rhinovirus, which commonly circulate at the end of the summer or in the fall. To further explore this, a negative regression model was run, with the number of specimens tested for other non-influenza respiratory viruses used as the outcome. Similarly, an inverse relationship between both AH and temperature and the number of specimens tested was found (data not shown).

Second, the seasonality of influenza A and B viruses is often different in most temperate climates; hence, we examined the association of each virus with environmental factors separately. Third, we constructed two linear and nonlinear negative regression models and obtained consistent results. Separating community-based specimens from those obtained during outbreaks is also a novel approach; the latter may be more indicative of nosocomial transmission rather than climactic factors. Finally, we considered both AH and RH in the model and accounted for the collinearity of AH with temperature.

In summary, our study found a negative association between both AH and temperature and influenza A and B virus occurrence. An increase in RH was associated with an increase in influenza A virus detection (which might have been influenced by indoor heating during winter) and a decrease in influenza B virus detection. Temperature fluctuation was associated with increased influenza B virus activity. Influenza virus was more likely detected in patients younger than 65 years of age but older than 1 year of age. Influenza A or B virus was less likely to be detected from community patients than from specimens tested as part of an outbreak investigation, which may also be reflective of nosocomial transmission. No effect of WS was found on either influenza A or B virus detection. The nonlinear nature of the relationship between influenza virus detection and environmental factors, particularly influenza A virus detection with temperature and influenza B virus detection with AH, RH, and temperature, could explain the complexity of and variation between influenza A and B viruses. Finally, in this study we report an association, but this ecologic study was unable to establish a cause-effect relationship. Based on the agreement between the results of the linear and nonlinear models, AH had a stronger effect on all influenza virus counts than RH. Unlike RH, which measures the air saturation point of water and varies by indoor versus outdoor location during the winter (the season of influenza activity in temperate climates), AH measures the actual amount of water in the air, regardless of temperature, and is consistently low indoors and outdoors during the winter (30). That might explain the consistent effect of AH on both influenza virus types and the strength of the association found in this study.

### Conclusion.

Predicting influenza activity is important for the timing of implementation of disease prevention and control measures as well as for resource allocation. Environmental factors may influence influenza A and B viruses differently. In the Toronto area, low AH and temperature are associated with seasonal influenza activity.

## MATERIALS AND METHODS

Environmental data were obtained in the Toronto area of Ontario, Canada, during the study period from 1 January 2010 to 31 December 2015 and were linked with each corresponding patient’s influenza virus testing data obtained at the same locality and during the same time period.

### Environmental data.

Hourly environmental data (RH, temperature, and WS) recorded at four meteorological stations (located at Pearson Airport and in Toronto City, Toronto Island, and Buttonville) in the selected area and during the period of study were obtained from Environment Canada (EC) in May 2016. These hourly data were transformed to daily, weekly, and monthly medians for each station. For the purpose of this study, RH was defined as a ratio of the partial water vapor pressure to the saturation water vapor pressure at the same temperature and was expressed as a percentage (31). AH was defined as the weight of water vapor per unit volume of air and was expressed as the number of grams per cubic meter. Since AH data were not available from EC, the following formula was used to calculate AH, based on the available temperature (*T*) and RH (32): AH = {6.112 × *e*^[(17.67 ×^ *^T^*^)/(^*^T^*^ ± 243.5)]^ × RH × 2.1674}/(273.15 + *T*).

Daily temperature fluctuation was defined as the difference in the median daily temperatures between the exposure day and the previous day and is expressed in degrees Celsius. Temperature fluctuation was included in the model to understand the role of temperature change on the number of positive influenza virus test results.

### Laboratory influenza virus testing data.

The majority of testing for influenza virus in the province of Ontario is conducted at the Public Health Ontario Laboratory (PHOL). For the purpose of this study, the laboratory-confirmed detection of influenza virus in respiratory specimens at PHOL was used as the measurement of influenza activity. Influenza virus testing data were extracted from the laboratory information management system (LIMS) of PHOL for the selected location and for the study period. Each patient’s location was determined by looking up the first three digits of the postal code of either the patient’s residence or the submitter’s address.

Only patients who resided in the Toronto area and for whom environmental data were available were included in this study.

The PHOL data included log-in date (the date that the specimen was received at PHOL), the patient’s and/or submitter’s postal code, the patient’s age, and outbreak status (indicating whether a specimen was submitted as part of an outbreak investigation, i.e., whether the specimen was assigned an outbreak number). The source of these data elements was from information provided on PHOL’s laboratory requisition forms.

Testing for influenza A and B viruses was performed by real-time reverse transcriptase PCR (rRT-PCR), viral multiplex PCR, and/or viral culture. Subtyping was performed for more than half of the specimens included in this study via influenza A virus subtype testing or viral multiplex PCR. Results were considered positive for influenza virus if any of the test methods showed positive results. The exposure date was used to link environmental data with laboratory influenza virus testing data. Two days (range, 1 to 3 days) has been reported to be the average incubation period of influenza virus infection (33). It is known from PHOL data that the median duration between symptom onset and specimen collection and between specimen collection and the log-in date is 2 days each, while the ranges vary from 0 to 30 and 0 to 28 days, respectively (data derived in a look back at PHOL respiratory surveillance data for the same time period). Therefore, considering the observations described above, the exposure date was defined as the 6th day preceding the log-in date. The exposure week and month were also calculated on the basis of the exposure date. Finally, all results were collated by week and outbreak status.

### Data analysis.

Descriptive data analyses were performed to characterize environmental factors (AH, RH, WS, temperature, and temperature fluctuation) in the study area by different time intervals (day, week, and month). Weekly averages were used for the multivariate analysis. Pearson correlation analyses were performed to assess the correlations within the various environmental factors and between the environmental factors and the number of samples with influenza viruses overall and with influenza A and influenza B viruses. Correlation analyses were also performed among the influenza A virus-positive specimens, with subtype testing being completed to examine the association of environmental factors with the influenza A/H3N2 virus subtype. Two negative binomial regression models were constructed to explore the relationship between environmental factors as independent variables and the number of specimens with influenza viruses overall and with influenza A and influenza B viruses separately as dependent variables. The number of specimens tested was used as the offset. A separate model was performed to examine the association of environmental factors with the presence of the influenza A/H3N2 virus subtype using all subtyped specimens as the offset. Other variables were not included in this model as the data set was restricted only to influenza A virus-positive specimens with which subtype testing had been completed.

The first model (the AH linear model) was adjusted for AH, temperature fluctuation, WS, patient age, and outbreak status. Temperature was not included in this model, as it was found to be highly correlated with AH. Since AH is a function of RH and temperature, these parameters can be traded; therefore, in the second model, AH was replaced by the last two. This simplified the equation and allowed both examination of the relation between temperature and influenza virus counts and comparison of the magnitude of the associations between AH and RH and influenza virus counts. Specifically, the second linear model (the RH linear model) was adjusted by RH, temperature, temperature fluctuation, WS, patient age, and outbreak status. In addition, an interaction term between temperature and temperature fluctuation was added to both models to investigate the possible effect modification of temperature fluctuation and temperature since the effect of a temperature decrease might be different between warmer and colder temperatures.

The models described above assumed a linear relationship between environmental exposures (AH, RH, and temperature) and influenza activity; however, such restrictions might overlook important nonlinear relationships. Therefore, the linear assumption was relaxed for both models by including restricted cubic spline terms. Specifically, the AH spline model explored the nonlinearity between AH and influenza outcomes, while the RH spline model looked at the nonlinearity of RH and temperature on the influenza outcomes. Both models were adjusted for all other variables included in the linear negative regression models. Interaction terms were excluded from the models since they were found to be neither statistically nor clinically significant.
